# A systematic review: what factors predict Post-Traumatic Stress Symptoms in ambulance personnel?

**DOI:** 10.29045/14784726.2021.3.5.4.18

**Published:** 2021-03-01

**Authors:** Damian Brooks, Rachel Brooks

**Affiliations:** Ridings Medical Group; Humber Teaching NHS Foundation Trust

**Keywords:** ambulance personnel, paramedic, PTSD

## Abstract

**Background:**

Ambulance personnel are frequently exposed to traumatic accidents, which makes them a high risk for poor mental health. High rates of Post-Traumatic Stress Symptoms (PTSS) have been found within ambulance personnel samples but no review has been completed to examine the factors that may be implicated in the development of these symptoms. This literature review provides an overview of the factors that predict PTSS in ambulance personnel.

**Methods:**

A systematic search strategy was conducted in April 2020 across the following four databases: PsycINFO, PsycARTICLES, MEDLINE and Cumulative Index to Nursing and Allied Health Literature (CINAHL).

**Results:**

Eighteen papers were included in this review, and the predictive factors examined were grouped into four categories: coping style, personal factors, environmental factors and organisational factors.

**Conclusions:**

An array of factors across the four categories were implicated in the development of PTSS, but these tended to be indicated in only one or two papers. Evidence was found to suggest that dysfunctional coping styles, reduced levels of some personality traits, proximity and nature of the critical incident and high levels of organisation stress can all lead to PTSS. Further research is needed to support the reliability of findings.

## Introduction

Front line ambulance personnel are frequently presented and involved with high stress situations, such as severe life-threatening diseases or road traffic collisions, and, as such, regular exposure to traumatic incidents is considered part of the job (McFarlane et al., 2009). These incidents require the clinician involved to make quick decisions in complex circumstances, and in many cases without support from other professionals. This, combined with multiple organisational demands such as shift work, disturbed sleeping patterns and being expected to manage difficult jobs and workloads, makes ambulance personnel a high risk demographic for poor mental health (Levin et al., 2014).

One element of mental health which is particular pertinent to ambulance personnel is Post Traumatic Stress Disorder (PTSD), which is commonly characterised by a response to a stressor/trauma that includes: intrusive symptoms such as flashbacks or nightmares; avoidance; and negative alterations in mood and cognition (Carmassi et al., 2016). An examination of the prevalence of mental health problems in the United Kingdom by Bennett et al. (2004) suggested that PTSD rates are as high as 20–22% in pre-hospital care workers.

When this statistic is compared against the prevalence of PTSD found within the general population, which is 8.8% (Propper et al., 2005), it is apparent that ambulance personnel are a higher risk group in relation to the development of PTSD. Furthermore, there is a need to understand the factors that predict PTSD in ambulance personnel in order to better support those at risk of suffering or who are already suffering with PTSD. Without this understanding and support, ambulance personnel will likely continue to have a higher rate of early retirement due to physical and mental ill health than any other healthcare staff or professional (Rodgers, 1998).

An initial scoping search highlighted that there has not been a literature review conducted in this area, and therefore this systematic literature review aims to evaluate the factors that predict PTSD symptoms in ambulance personnel.

## Method

The PRISMA reporting method was used throughout this review (Moher et al., 2009). The patient group (P) included ambulance personnel, the intervention (I) was predictors of PTSD, with no comparator (C) being used to achieve the outcome (O) which was a review of predictors of PTSD in ambulance personnel.

A systematic search of the literature was conducted across four databases: PsycINFO, PsycARTICLES, MEDLINE and Cumulative Index to Nursing and Allied Health Literature (CINAHL). These databases were chosen to cover a wide array of both health and psychological literature. No date ranges were set. Two limiters were applied to the search, namely that papers had to be (1) published in the English language to avoid translation errors, and (2) peer reviewed in order to try and ensure the research was of good quality.

Search terms spanned the following two categories: (1) ‘PTSD’ OR ‘Post traumatic stress disorder*’ OR ‘Post-traumatic stress disorder*’ OR ‘Posttraumatic stress disorder*’, and (2) ‘Paramedic*’ OR ‘EMS’ OR ‘emergency medical service*’ or ‘prehospital’ or ‘pre-hospital’ or ‘ambulance*’ or ‘emergency medical technician*’ or ‘EMT’ or ‘ECA’ OR ‘emergency care assistant*’. The truncation (*) was used to capture various endings of the same word. Additional criteria were that the text had to be in the English language and published in a peer-reviewed journal, and that the sample had to be ambulance personnel only and not those employed in other roles such as nursing, police or firefighting, to ensure that the data was strictly relevant to ambulance personnel only. Case studies or literature reviews were excluded, as were student paramedics or combat paramedics due to these being seen as distinct job roles that differ from ‘typical’ expectations of ambulance personnel.

All abstracts identified were reviewed by the primary author (DB), and reference lists were also examined. A shortlist of abstracts was used to guide full-text reviews, and papers were included if they had examined a factor in relation to Post-Traumatic Stress Symptoms (PTSS) using only ambulance personnel. For this reason, several papers were excluded because they were deemed to be ‘irrelevant’ as they did not meet these inclusion criteria and examine a predictor of PTSS. A number of papers were also excluded as they included ‘mixed samples’ which contained excluded job roles – for example, both paramedics and student paramedics.

### Quality review

The methodological quality of each paper was checked using the Mixed Methods Appraisal Tool (MMAT; Hong et al., 2018). The MMAT was chosen due to its ability to be used across different methodologies – this made it suitable for the current review as the 18 selected papers consist of quantitative and mixed methods designs. In order to use the MMAT, a paper must pass two screening questions, followed by a further five questions, making a score of five the best a paper could achieve. In order to assess the inter-rater reliability of the scoring, a random selection of papers (n = 8) were marked independently by both authors, to check for consistency of scoring. The same scores were given to each paper that was randomly selected.

## Results

**Figure 1:**
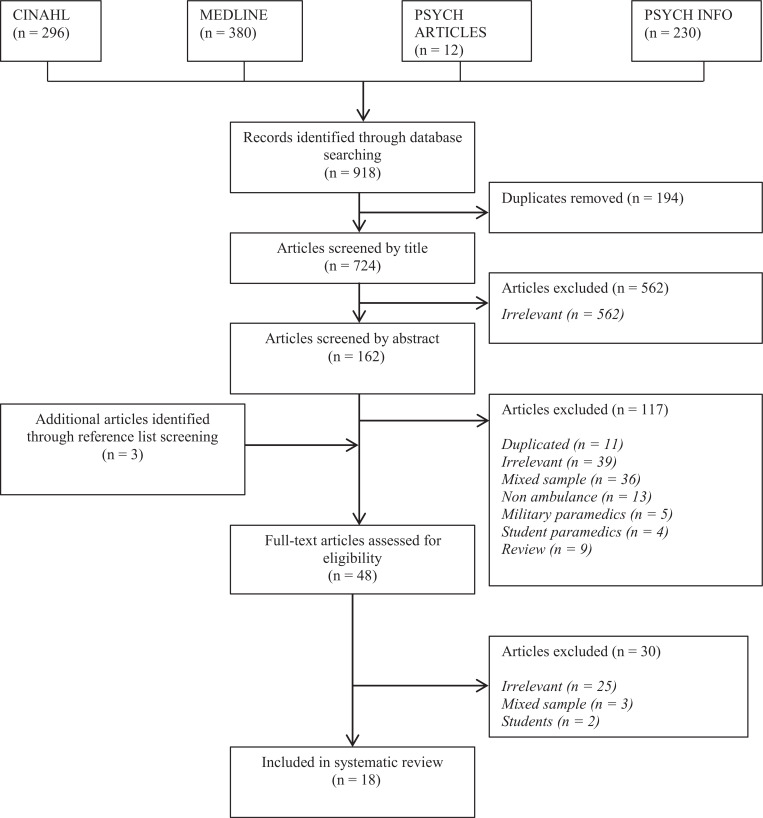
Figure 1. Results of literature review and selection process.

The systematic literature review search was carried out on 16 April 2020. In total 918 papers were identified, which were examined by the primary author (DB). A total of 18 papers were considered to be suitable for inclusion in this review. This process is highlighted in Figure 1. All included papers were integrated into a table pre-designed to gain the following information: year of publication, study design, number of participants, data collection methods, data analysis methods and results. A table of included papers and key details is included as a supplementary file (Supplementary 1).

### Overview of included studies

Eighteen papers were included in this review, aimed at exploring the factors that predict PTSS/PTSD. From examining the included papers, it was felt that they could be separated into four groups of predictors: (1) coping style, (2) personal factors, (3) environmental factors and (4) organisational factors. Accordingly, the results and subsequent discussions will be presented in these four groups. All papers were assessed for quality (see Supplementary 1 for individual MMAT scores) and each achieved a score of 4/5 or 5/5, suggesting a good quality of research and low risk of bias. No papers were excluded based on quality due to this.

### Coping style

Clohessey and Ehlers (1999) aimed to investigate the response to intrusive memories, and if ambulance service workers’ coping styles can influence the development of PTSD. They found that workers who dwelled upon intrusive memories and thoughts from certain call-outs, commonly known as ‘jobs’, were significantly at risk of increased severity of PTSD (*p* < 0.01) and of significantly lower general health scores (*p* < 0.01). Correspondingly, Kucmin et al. (2018) aimed to explore if coping styles or dispositional optimism could predict PTSD symptoms and severity. The study found that an emotion-focused coping style, which involves trying to reduce negative emotional responses, was a significant predictor in PTSD (*p* < 0.05), and that dispositional optimism can mediate this relationship.

Similarly, Köhler et al. (2019) investigated whether dysfunctional disclosure, such as avoidance or reluctance to disclose or repeatedly disclosing, can mediate the relationship between negative appraisals and PTSD. The results from this study highlighted that PTSD symptom severity between individuals with PTSD and those without did not differ in variables of trauma exposure, but was significant (*p* < 0.001) with respect to PTSD severity dependent on peri- and post-trauma processing and communication behaviour. It also indicated that the endorsement of negative trauma-related appraisals or thoughts can be a viable predictor in the development of PTSD (*p* < 0.001).

Another paper that considered disclosure and communication post critical incident was Halpern et al. (2012), who examined the impact that two coping strategies had on current PTSD symptoms, either (1) identifying emotions, or (2) describing and expressing emotions. They found that alexithymia (difficulty expressing emotions) and all its components were associated with virtually all current symptoms (*p* < 0.05), whereas voluntary emotional expression was unrelated to current symptoms. They also found that greater emotional expression led to greater perceived helpfulness (*p* < 0.001).

Additional evidence comes from Kerai et al. (2017), who aimed to assess symptoms of PTSD and whether specific predictors could be identified among emergency medical staff (EMS). They found that people who use dysfunctional coping strategies were shown to have an increased severity in PTSD (*p* < 0.05). Subjects who initially scored high in anxiety and depression were at higher risk of developing PTSD (*p* < 0.05).

Further dysfunctional coping styles were identified by Donnelly (2012), who explored the difference between different types of work-related stress and alcohol use and if these influence the development of PTSD. The study found that staff suffering with high post-traumatic stress levels used alcohol more frequently (*p* < 0.01). Similarly, Van der Velden et al. (2008) explored whether smoking could be considered a risk factor in developing PTSD among emergency medical responders (EMRs), and found that cigarette consumption could independently predict intrusions (*p* = < 0.019), avoidance (*p* = < 0.031), hostility (*p* = < 0.012) and depression (*p* = < 0.032).

### Personal (age, length of service, grit, resilience and sense of coherence)

Streb et al. (2014) conducted a study aimed at examining sense of coherence (SOC) and resilience to explore if these factors are associated with PTSD severity. They found that a decrease in resilience and SOC was significantly correlated with severity of PTSD (*p* < 0.01), as was age (*p* < 0.05), although length of service was not indicated. Another paper that examined SOC was Jonsson et al. (2003), who found that a lower SOC and the increased amount of time in service were both valid factors (*p* < 0.05) in the development of PTSD.

In a different study, Bennett et al. (2005) aimed to identify incident factors which lead to emotional distress. The results showed that men were significantly more likely to develop PTSD than women (*p* < 0.05), and that PTSD severity could be predicted by the frequency of attending traumatic jobs, length of service and dissociation at the time of the event. Interestingly, no differences were found according to clinical grade.

Additionally, Musso et al. (2019) investigated the relationship between grit and resilience among EMS workers. They found that post-traumatic checklist scores were significantly negatively correlated with the Grit scale score (*p* < 0.001), and also that lower grit scores were significant predictors of denial and substance use.

Finally, Halpern et al. (2012) looked at the impact of peri-trauma (occurring during and/or immediately after a traumatic event) factors on post-traumatic symptoms. This study investigated whether certain characteristics of critical incidents lead to distress and impaired functioning. They reported that all three characteristic domains (situational, personal and systemic) had a negative effect on recovery and emotional symptoms post trauma (*p* < 0.001), and that there is a strong relationship between these domains and current PTSD symptoms.

### Environmental (type of job, abuse, aggression, distance to incident)

A study carried out by Michael et al. (2016) aimed to investigate the differences in post-traumatic cognitions and how intrusions are dealt with in relation to PTSD symptoms after direct and indirect threats to workers. The results highlighted that 34% of paramedics experienced indirect threats while 66% reported direct threats. Significantly more men (71%) reported direct threats (*p* < 0.001). The direct threat group had more chance of developing partial or full PTSD when compared against the indirect group (*p* < 0.017). Additionally, direct threats were more associated with dysfunctional post-traumatic cognitions.

Similar findings were discussed by Misra et al. (2009), who investigated risk factors in London Ambulance Service (LAS) staff. It was found that those responding closest to the incident site showed a significant risk of developing PTSD (*p* < 0.05), showing that proximity is a PTSD predictor.

Khashaba et al. (2014) aimed to assess the levels of psychosocial stress and related hazards such as burnout, depression and PTSD among EMRs. They reported an increased risk of PTSD for those who had higher stress levels from deaths of colleagues, exposure to verbal or physical assault or dealing with psychiatric-related cases. They also reported that EMRs were significantly more likely to develop PTSD than a comparative group of emergency call handlers and dispatchers.

### Organisational (workplace stressors/policies/procedures)

Donnelly et al. (2016) investigated the relationship between workplace stressors and PTSD. The results indicated that PTSD was significantly correlated with operational stress (*p* < 0.001), organisational stress (*p* < 0.001) and critical incident stress (*p* < 0.001). Results revealed that chronic operational stress was a significant independent predictor of PTSS (*p* < 0.001) and in combination with critical incident stress (*p* < 0.01). Paramedics also reported a higher preference for receiving support from a work partner, friend or family member than from other sources (*p* < 0.001).

Additionally, Halpern et al. (2014) aimed to examine the relationship between a downtime period and long-term emotional symptoms. The results identified that receiving any downtime was associated with significantly lower depressive symptoms (*p* < 0.008). Downtime was not significantly associated with post-traumatic symptoms (*p* = 0.48), burnout (*p* = 0.12) or somatic symptoms (*p* = 0.07). Therefore, there seems to be a characteristic of downtime that does not mitigate PTSD but does mitigate depression.

## Discussion

### Overview of research findings

The aim of this systematic literature review was to investigate what factors predict PTSD/PTSS in ambulance personnel. Results were split into four groups of factors, although there was overlap. Although an array of factors were found in this review, most of these were only implicated in one or two papers, and this means that the following discussion points and conclusion should be interpreted with caution.

### Coping styles

Although a number of different negative coping styles/responses were highlighted, few papers discussed the same coping style, making it difficult to make clear conclusions. What seems clear from the research is that how a person responds to a critical incident will likely influence their probability of developing PTSD symptomology. These factors seem to span (1) how a person thinks or feels about the critical incident, (2) how a person talks/communicates about the critical incident and (3) what a person does to manage distress in the longer term.

Furthermore, the evidence suggests that adopting a negative coping style, such as rumination on thoughts (Clohessy & Ehlers, 1999), dysfunctional disclosure (Kohler et al., 2019) or increased alcohol use (Donnelly, 2012), seems to lead to an increase in PTSS. It is worth keeping in mind that many of the coping styles discussed could be viewed as symptoms of PTSD; for example, dwelling on intrusive memories (Clohessy & Ehlers, 1999) is a hallmark feature of PTSD (Carmassi et al., 2016). This inevitably leads to a ‘chicken or egg’ dilemma, where it becomes difficult to ascertain if the coping styles discussed are predictors or consequences of PTSD.

### Personal

Of the five papers reported in this section, a range of personal factors were implicated, including SOC, resilience, grit and length of service. Again, each factor was only implicated in one or two papers, making clear conclusions difficult. However, it is apparent that across the personality traits investigated, PTSS was significantly predicted by the lower the level of positive trait found – for example, lower resilience is suggested to lead to increased PTSS (Streb et al., 2014). The evidence for the predictive factor of length of service was mixed, and only one paper indicated that gender was a predictive factor of PTSS (Bennett et al., 2005).

It should be mentioned that the use of quantitative measures to assess personality traits has attracted criticism, mainly due to the fact that these measures reduce complex and comprehensive aspects of the human mind and personality to numbers. Therefore, the conclusions made from research papers that employ this type of methodology should be interpreted with caution, as they fail to take into account the fluidity of personality factors, which can be influenced by many life/personal circumstances not controlled for by this study. It is also likely that an individual living with PTSS may experience a decrease in positive personality traits – for example, somebody experiencing frequent flashbacks or nightmares may find that their levels of resilience decrease. Furthermore, the personality factors indicated may also be a consequence of PTSS.

### Environmental

Of the three papers that investigated environmental factors as predictors of PTSS, all proposed evidence that the type of job/incident impacts the prevalence of PTSS. Being exposed to a traumatic job was a major factor, but factors such as proximity (Misra et al., 2009), frequency (Khashaba et al., 2014) and a threat being direct (Michael et al., 2016) were also all found to predict PTSS symptoms. This information is particularly useful for those in senior positions within ambulance services to hold in mind, as it may help identify those at a higher risk of developing PTSS, and who would likely benefit from support and debriefing or downtime following such incidents.

### Organisational

The final two papers in this review focused on organisational factors as predictors of PTSD, with varying aims. The finding that chronic operational stress predicts PTSD (Donnelly et al., 2016) is an unsurprising finding at face value, but has interesting implications when considered with the rest of the findings of the review. It would be reductionist to assume that chronic organisational stress is solely responsible for predicting PTSD, especially as PTSD needs a traumatic event/stressor to develop, but it does highlight that there may be a relationship between organisational stress and how ambulance personnel respond to / manage traumatic jobs. For example, it may be that staff shortages lead to an ambulance crew responding independently to a job that would otherwise require backup, making the job potentially more challenging and/or traumatic. Equally, the same example could lead to an individual feeling as though their treatment approach was sub-par, and therefore they may experience a low SOC.

Interestingly, this finding has implications for Halpern et al.’s (2014) research, which highlighted that downtime significantly reduced depressive symptoms but not PTSD symptoms. Although there was no direct link between downtime and PTSD, it is worth considering that an indirect link to PTSD that could exist. For example, downtime was found to significantly mitigate depressive symptoms, which could otherwise lead to staff absences and therefore organisational stress, which was indicated in the prediction of PTSD. This example alone highlights that indirect predictors of PTSD should be considered further.

### Methodological limitations and future research

The two biggest limitations of this research relate to the way in which it was conducted, and the classification around PTSD. Firstly, as previously mentioned, is that the sole use of questionnaires is a reductionist approach that reduces complex relationships to numbers. Few papers used a mixed methods approach, and even within these papers the qualitative elements were small sections on questionnaires rather than in-depth explorations of ambulance personnel opinions or experiences of some of the factors thought to predict PTSD symptomology. Future research should expand on the use of questionnaires to gain further understanding of the experiences of ambulance staff, potentially by using semi-structured interviews or open-ended questions on surveys to gain richer data.

Following this, there were differences between papers on the tools used to measure symptomology and whether this was termed PTSD, PTSS or trauma symptoms. According to the Diagnostic and Statistical Manual 5 (Houts, 2002), PTSD can only be diagnosed following a thorough assessment by a registered psychiatrist, and therefore the questionnaires used in many of the papers should only be considered as screening, not diagnostic, tools. No clear conclusions can be made about the factors’ ability to predict PTSD; rather, they give an indication of the presence of symptoms associated with them. Other notable limitations of this review include the use of only four databases, and a lack of limiters regarding age and location of research.

## Conclusions

This literature review has implicated a range of factors in the development of PTSD symptoms, but unfortunately it fails to arrive at any clear conclusions about which may be more important in the development of PTSD symptomology. Due to a large number of factors being implicated, often across just one paper, further studies demonstrating the same findings would be needed to improve reliability and validity. That being said, there is evidence that dysfunctional coping styles, reduced levels of some personality traits, proximity and nature of the critical incident and high levels of organisation stress can all lead to PTSS. Future research is needed to strengthen the evidence base for each group of factors, and to consider that many of these factors are likely to be linked and intertwined in a complex relationship, rather than existing in isolation.

## Author contributions

DB was the lead author and subsequently led on the planning of the project, completing the literature search, analysis of the data and write-up of the paper. RB was the supervisor for this project and had oversight and input on all areas of the research, with significant contributions to completing the literature search and analysis of the data. RB acts as the guarantor for this article.

## Conflict of interest

None declared.

## Ethics

Not required.

## Funding

None.
